# Early colposcopy in non-16/18 high-risk human papillomavirus positivity with normal cytology

**DOI:** 10.1590/1806-9282.20252178

**Published:** 2026-06-29

**Authors:** Elif Görkem Bademci, Didem Soysal, Mehtap Güneş, Ahmet Zengin

**Affiliations:** 1 Sancaktepe Şehit Prof. Dr. İlhan Varank Training and Research Hospital – Istanbul, Turkey.; 2 Marmara University, Faculty of Medicine – Istanbul, Turkey.; 3 Ümraniye Training and Research Hospital – Istanbul, Turkey.; 4 Tarsus State Hospital – Mersin, Turkey.

**Keywords:** Human papillomavirus, Colposcopy, Uterine cervical neoplasms, Papillomavirus infections

## Abstract

**OBJECTIVE:**

Very few clinical studies have been done to evaluate the potential clinical benefit of utilizing early colposcopy in women with normal Pap smear cytology but positive for high-risk human papillomavirus types other than human papillomavirus 16 or human papillomavirus 18 in a screening population for cervical cancer. The aim of this study was to compare two follow-up strategies and assess the need for colposcopy at the initial visit, based on cytology, human papillomavirus testing, and colposcopic findings at 1 year, among study participants.

**METHODS:**

In total, 122 women who had negative Pap smears and were positive for high-risk types of human papillomavirus (not human papillomavirus 16/18) were randomly allocated to two groups via simple computer-generated randomization. Notably, one group had a colposcopic examination when they were first diagnosed (61), and the other group had standard Pap/human papillomavirus follow-up without a colposcopic examination (61). All women in both groups had repeat Pap and human papillomavirus testing and a colposcopic examination after 1 year.

**RESULTS:**

Rates of loop electrosurgical excision procedure application were similar between groups (11.5 and 8.2%; p=0.343). So early colposcopy has enabled the treatment of high-grade squamous intraepithelial lesion lesions without increasing the number of loop electrosurgical excision procedures.

**CONCLUSION:**

In women who had negative cytology yet positive high-risk human papillomavirus types other than human papillomavirus 16/18, there was no statistically significant difference in either high-risk human papillomavirus persistence, abnormal cytology rates, or loop electrosurgical excision procedure requirements at a 1-year follow-up after having their colposcopy performed early vs. delayed assessments. This suggests that these patients can be appropriately managed with risk-based follow-up care.

## INTRODUCTION

Cervical cancer is one of the deadliest cancers for women globally. Based on GLOBOCAN 2022 data, cervical cancer is the 4th leading cause of cancer in women, with an enormous amount of morbidity and mortality from gynecologic cancers. The annual worldwide incidence of cervical cancer is about 0.01%^1^. In Turkey, the incidence of cervical cancer was reported by the General Directorate of Public Health as 4.5 per 100,000 women in 2025^2^. Although there has been a declining trend in the incidence rate of cervical cancer as a result of better access to effective screening programs and increased use of the HPV vaccine in developed nations, cervical cancer remains a significant public health dilemma worldwide, particularly in developing nations.

HPV (Human papillomavirus) is primarily responsible for the incidence of cervical cancer. Over 200 types of HPV have been identified worldwide to date; some are called low-risk, while others are considered high-risk and can cause cancer. In terms of risk from HPV, there are two principal groups: The two highest-risk types (HPV 16 and HPV 18) have been extensively researched, but many other types in the "High-Risk" category also significantly contribute to the development of cervical cancer, e.g., HPV 31, HPV 33, HPV 35, HPV 39, HPV 45, HPV 51, HPV 52, HPV 56, HPV 58, HPV 59, HPV 66, HPV 68, and HPV 73^3^. The incidence of HPV varies by geographic location, with the highest rates in low- and middle-income regions^4^. A recent meta-analysis published that included 78 studies of women with normal cytology showed that HPV prevalence is 22.1% in Africa, 20.4% in Central America/Mexico, 11.3% in North America, 8.1% in Europe, and 8.0% in Asia^5^.

The study performed in Turkey as part of a national screening project revealed that HPV positivity among participating females was 4.39%. Almost 70% of HPV-positive individuals had normal cytological results, while 14% had abnormal results. Additionally, among the total HPV-positive population with normal cytology, the prevalence of HPV types 16, 18, and 31 or 51 was 28.1, 6.7, and 25.1%, respectively^3^. There is, therefore, a large population of HPV-positive females with normal cytology and a significant genetic risk factor for developing cervical cancer.

Worldwide, the combination of cytology and testing for the presence of HPV (i.e., HPV cotesting) in females has been recommended for cervical cancer screening by numerous national and international organizations. Risk-based screening algorithms developed by these organizations aim to prevent patients from undergoing unnecessary invasive procedures and to ensure the appropriate use of healthcare resources. On the other hand, advances in DNA amplification methods have led to new screening methods for detecting human papillomavirus (HPV) genetic material, specifically high-risk HPV (hrHPV)^6^. Urine and vaginal self-sampling has begun to be incorporated into HPV screening programs due to its high sensitivity, high negative predictive value, and ease of use^7^.

Direct colposcopy is not recommended for HPV-positive women with normal cytology, provided they are positive for a high-risk strain (other than HPV 16 or 18); rather, repeat cytology and HPV testing should occur 1 year later. If they continue to test positive for HPV, or develop cytological abnormalities, colposcopy is indicated^8^. This approach has significant advantages in preventing unnecessary colposcopic and biopsy procedures. Nonetheless, recent research indicates that other types of HPV can also be considered high risk for developing premalignant lesions in some populations of women. Although there is ongoing debate about how best to manage women diagnosed with positive hrHPV but without abnormal cytology, many experts agree that it would be beneficial to perform early colposcopic evaluations on women with positive hrHPV test results to detect possible high-grade lesions sooner rather than later; however, there have not been too many studies conducted demonstrating the effectiveness of this approach clinically or materially. The purpose of this research project, therefore, is to evaluate whether performing early colposcopies is necessary for women who have had an abnormal Pap test but are positive for hrHPV (not subtypes 16 or 18). To accomplish this objective, we will compare the cytological (i.e., the results from a Pap test), virological (i.e., the results from an hrHPV test), and histopathological (i.e., the pathology of a biopsy or excised specimen) results after a 1-year follow-up period between those women who were seen by their physicians at an early clinic for an early colposcopic examination and those women who only had follow-up physicians through standard follow-up procedures utilized for all patients with negative hrHPV test results. The ultimate goal of this research study is to provide information to help physicians determine the best clinical management approach for these patients.

## METHODS

### Study design and population

This is a prospective, analytical cohort study conducted at the Gynecologic Oncology Clinic of a tertiary referral center, with the study period spanning 2021 to 2022. The presence of an abnormal colposcopic biopsy at 1-year follow-up was defined as the study's primary outcome. The initial sample size estimation was not performed at the start of the study, and a total of 122 participants who met the criteria were enrolled during the study period. Based on the observed rates of an abnormal colposcopic biopsy between the two groups (5% of group 1 vs. 16% of group 2), it has been estimated that a total of 242 participants would have been needed for adequate power (121 in each group), given an α-level of 0.05 and 80% power. As such, this study should be viewed as a pilot study with very limited statistical power, particularly for rare outcomes. A total of 122 non-pregnant female participants, 21–65 years of age, with negative pregnancy tests and a histologically normal cervical cytology with positive hrHPV other than types 16 and 18, were included in this study after written informed consent from each participant was obtained. The study protocol was approved by the ethics committee of Umraniye Education and Research Hospital (approval no. 22484).

The study's exclusion criteria included history of prior hysterectomy, history of any current or past pregnancy, presence of any suspicious lesions on the cervix found at the time of speculum examination, and history of cervical conization. All participants had demographic/obstetric data collected (age, number of pregnancies, parity, smoking status).

### Grouping and procedures

At the beginning of the study, participants were randomly assigned to one of two groups using a computer-generated simple randomization method, with a 1:1 ratio and equal numbers of patients in each group. The randomization list was created by an independent researcher who was not involved with the study, and group assignments were made using sealed opaque envelopes. This prevented researchers from influencing patient selection.

For purposes of analysis, the participants were divided into two groups:

Group 1—Patients who had colposcopic examinations at their initial appointments (n=61).

Group 2—Patients who did not have colposcopic examinations at their initial appointments and received routine follow-up (n=61).

### Follow-up and diagnostic procedures

After 1 year, all participants will have had repeat Pap tests (Pap smear cytology) and HPV screening (both tests were run regardless of the participants’ initial management strategy) and had repeat colposcopic examinations (CE). The cytology tests (i.e., tests using cervical cytology specimens for Pap testing) were evaluated using the 2014 Bethesda System by experienced gynecological pathologists. The cervical cytology specimens were collected using the ThinPrep^®^ liquid-based cytology technology; HPV typing was performed for the 14 HPV types that carry a high risk for cervical cancer (HPV types 16, 18, 31, 33, 35, 39, 45, 51, 52, 56, 58, 59, 66, and 68) from the same cervical cell specimen (the sample had not been destroyed). All HPV+samples had a follow-up typing of HPV type 16 (HPV 16), as well as HPV type 18 and/or 45.

### Colposcopic assessment

All colposcopies were conducted by a single qualified investigator with experience in colposcopic research. At first, the cervix was cleaned with saline, then examined with a green filter to determine the vascular pattern at low magnification. The cervix was then treated with 3–5% acetic acid and examined at low and high magnification after 1 min. The cervix was then stained with 10% Lugol's iodine; areas that did not stain with iodine were noted.

Targeted biopsies were performed on areas of acetowhite or iodine-negative abnormality that had punctuation, displayed a mosaic pattern, contained erosion, or had evidence of leukoplakia or atypical blood vessels. All biopsies were fixed in formalin and sent for histopathological analysis; no biopsy was performed if no abnormal findings were present. The results of the colposcopic biopsy were classified as normal, low-grade squamous intraepithelial lesion (LSIL), or high-grade squamous intraepithelial lesion (HSIL).

The results from the first application of the research and from the current application were recorded in the same manner and compared to determine whether any variations existed. The statistical software package SPSS v20 was used to conduct statistical analyses. Chi-square tests and Fisher's exact tests were performed where appropriate to assess statistical differences among categorical variables; t-tests were used to compare quantitative data. Two-tailed p<0.05 values indicated significant differences among the respective groups.

## RESULTS

A total of 122 patients were studied: 61 had a colposcopy at their first visit and 61 were simply monitored after that initial visit (without a colposcopy).

The mean age of patients in the colposcopy group was 45.6 years (±8.5) and 43.8 years (±8.6) in those who had not had a colposcopy; thus, there was no significant difference between the two groups (p=0.256). In addition, there were no statistically significant differences in the number of children born (parity); the colposcopy group had an average of 2.2 (±1.0) children; the other group had an average of 2.4 (±1.7) children (p=0.463). Smoking status (% [smoking]: colposcopy group 51% and the group without any colposcopy had 39%) (p=0.179). Lastly, no significant difference was found in the number of women capable of having children (% reproductive age: 80.3% for the group having a colposcopy vs. 73.8% for the group without a colposcopy) (p=0.389) (Table 1).

**Table 1:** Baseline demographic and clinical characteristics of the study groups.

Parameter	Initial colposcopy group (n=61)	Follow-up without initial colposcopy (n=61)	p-value
Age (years), mean±SD	45.6±8.5	43.8±8.6	0.256
Parity, mean±SD	2.2±1.0	2.4±1.7	0.463
Smoking, n (%)	31 (51%)	24 (39%)	0.179
Patients in reproductive period, n (%)	49 (80.3%)	45 (73.8%)	0.389
HPV positivity at 1 year, n (%)	16 (26%)	18 (29%)	0.840
Abnormal cytology at 1 year, n (%)	10 (16%)	12 (18%)	0.608
Abnormal colposcopic biopsy at 1 year, n (%)	3 (5%)	10 (16%)	**0.008** *
LEEP performed during study period, n (%)	7 (11.5%)	5 (8.2%)	0.343

* Values are presented as mean±standard deviation (SD) or n (%).

LEEP: loop electrosurgical excision procedure; SD: standard deviation; HPV: human papillomavirus. Bold values indicate statistically significant results (p<0.05).

Follow-up data collected 1 year after the initial examination showed that hrHPV positivity rates (persistent) did not differ significantly between the colposcopy and non-colposcopy groups (26 vs. 29%, respectively). There was also no significant difference in the rates of abnormal cytology between the two groups (16% in the colposcopy group vs. 18% in the non-colposcopy group). Additionally, when reviewed 1 year after their initial visit, there was a significant difference in the results of the colposcopically guided biopsies from the two groups. Specifically, after the initial colposcopic exam, 95% of women who underwent a second colposcopy had normal histology, 5% had LSIL, and no HSIL lesions were found among women who had previously received a colposcopy. Among the women who did not receive a colposcopic exam, 83% had normal histology, 8% had LSIL, and 8% had HSIL. The difference in the frequency of abnormal colposcopic biopsies between the colposcopy group and the non-colposcopy group was statistically significant (p=0.008) (Table 2). However, it is not surprising that treating lesions immediately will result in fewer lesions 1 year later.

**Table 2:** Comparison of histopathological outcomes between groups.

Outcome		Initial colposcopy group (n=61)	Follow-up without initial colposcopy (n=61)	p-value
Initial colposcopic biopsy results				
	Normal	50 (82%)	–	–
	LSIL	6 (9.8%)	–	–
	HSIL	5 (8.2%)	–	–
One-year follow-up colposcopic biopsy results				
	Normal	58 (95%)	52 (83%)	
	LSIL	3 (5%)	5 (8%)	
	HSIL	0	5 (8%)	**0.008**
	LEEP performed	7 (11.5%)	5 (8.2%)	0.343

Values are presented as n (%). LSIL: Low-grade Squamous Intraepithelial Lesion; HSIL: high-grade squamous intraepithelial lesion; LEEP: loop electrosurgical excision procedure. Bold values indicate statistically significant results (p<0.05).

A total of seven LEEP procedures were carried out (11.5% of the number of women in the colposcopy group), and five women underwent LEEP procedures from the non-colposcopy group (8.2% of the number of women in the non-colposcopy group). No significant difference existed between the two groups with regard to the number of LEEP procedures performed (p=0.343) (Table 2). So early colposcopy has enabled the treatment of HSIL lesions without increasing the number of LEEP procedures.

## DISCUSSION

Women infected with hrHPV types besides HPV 16 and 18 may also face an increased risk of developing cervical intraepithelial neoplasia (CIN) and invasive cervical cancer based on data from new studies. Although HPV 16 and 18 account for the majority of cases of cervical cancer, the role of other oncogenic hrHPV types, including HPV 31, 33, 45, 52, and 58, in the carcinogenic process of cervical cancer, has become more clearly defined^9,10^. Though no specific genotype has been definitively identified as associated with increased risk of HSIL, several studies indicate that among women with normal cytological findings who are positive for non-HPV-16/18 hrHPV genotypes, the risk of developing HSIL could be as high as 11.4%^11,12^. A few studies also suggest that, among non-HPV-16/18 hrHPV genotypes, some share similar risk for development of HSIL as those with HPV types 16 and 18^13^.

Cervical cancer is caused by HPV infection, the number one cause of cervical cancer that is easily preventable. The presence of HPV DNA has been detected in approximately 99.7% of cervical malignancies^14^. Of all the 200+ known HPV genotypes, HPV 16 and 18 are responsible for about 57 and 16% of cervical cancer cases, while the remaining oncogenic HPV types account for approximately one-fourth of worldwide cancer cases^15,16^. It appears that hrHPV genotypes, not only HPV types 16 and 18, could also play a major role in the development of cervical cancer; however, currently, there are still varying opinions on the appropriate follow-up protocol for patients with these types. A few specialists advocate earlier colposcopic evaluations to avoid missing high-grade lesions. Others, however, favor a more conservative approach using Pap cytology as well as repeat HPV testing for up to 6 months before performing invasive procedures^8^.

For decades, the Pap smear has been the main way to find out if someone has cervical cancer. But the rate at which this test can pick out pre-cancers of the cervix is extremely low in actuality. It is estimated that there is a 40% success rate for pre-cancer detection with Pap smears, but a 96% success rate for non-pre-cancerous conditions^17^. Repše-Fokter and coworkers found that almost 43% of the women with invasive cervical cancer were given a negative or low-grade Pap smear result from 1 to 40 months ago^13^. This proves that relying solely on Pap smears is inadequate for detecting pre-cancerous changes. Colposcopy can help find cervical pre-cancerous changes missed by Pap smears. Approximately 93% of cervical adenocarcinomas have HPV infection^18^.

Cytology showed an abnormal rate of 9% among all patients included in our study, whereas the abnormality rate for colposcopic biopsy results was ~15.3%. These findings indicate that cytology alone may not be sufficient to identify patients with high-grade lesions. In contrast, no statistically significant differences were found between the two management strategies evaluated in this study in hrHPV persistence, abnormal cytology, and LEEP application rates at 1-year follow-up. Based on these results, it appears that early colposcopy may not confer any additional benefits over the standard follow-up procedure with respect to short-term clinical outcomes.

The 2019 ASCCP guidelines recommend colposcopy for women who are hrHPV positive and have cytological abnormalities (≥ASC-US) or those women who are HPV 16 or 18 positive, regardless of cytology^8^. Conversely, women who are infected with hrHPV types other than HPV 16 and 18 and have normal cytology should have their colposcopy deferred and retested at 12 months. The findings of this study largely support this risk-based approach to management.

Nonetheless, only a small number of high-grade lesions were identified during follow-up of women who had not undergone colposcopy at the initial visit. Other research has presented similar findings. In their study, Madendağ et al. found that 12.4% of women with hrHPV types other than HPV 16 and 18 had HSIL, and 6.9% had negative initial cytology^17^. Bai et al. measured HSIL rates of 9.6 and 3.9% in non-16/18 hrHPV-positive women, even when initial cytology was normal^18^. In addition, studies have shown that specific hrHPV types, such as HPV 33 and HPV 31, may represent a greater risk of HSIL development than HPV 18^19,20^. Although the overall risk for hrHPV types occurs other than HPV 16 and 18, clinically relevant lesions can occur.

Our analysis showed that only a few patients were diagnosed with HSIL lesions during follow-up. Additionally, one patient from our non-colposcopy group was diagnosed with cervical adenocarcinoma after LEEP; this diagnosis should be interpreted with caution but highlights that our results do not support making definitive conclusions about the risk of invasive cancer based on this single case due to our limited sample size. Furthermore, because our study lacks sufficient statistical power to analyze rare outcomes, such as the incidence of cervical cancer, we cannot draw definitive conclusions based solely on this finding. In general, performing an early colposcopy does not provide a significant improvement in short-term outcomes (1 year post-colposcopy) compared with patients with normal cytology and hrHPV-negative results (excluding HPV 16 and 18). The results of our study suggest that performing an early colposcopy may allow for earlier detection of various lesions, but these patients will have the same management (treatment) outcome, whether or not they undergo an early colposcopy, over a 1-year follow-up period. Further research, including larger, multicenter, long-term studies, is needed to identify the most effective triage strategy for these patients.

More and more studies are documenting the clinical importance of hrHPV types that do not include HPV types 16 or 18. For example, previous cohort studies involving large numbers of subjects have suggested that infection by genotypes HPV 31, HPV 33, HPV 45, HPV 52, and HPV 58 may be important risk factors for the development of high-grade cervical lesions. A large-scale meta-analysis published in 2023 found that hrHPV types, excluding HPV type 16 and HPV type 18, represent significant risks for the development of HSIL or worse among women who have been infected with these virulent genotypes^21^. In a similar fashion, a systematic review conducted by Arbyn et al. in 2024 indicated that infection by means of hrHPV genotypes other than HPV types 16 or 18 was associated with a clinically significant risk of developing CIN HSIL or worse^22^.

A second article published in 2025 stated that the risk of developing HSIL or worse among women with normal cervical cytology and without hrHPV 16 or 18 infection is low; however, it is still present^23^. Also, when looking at the difference in risk associated with HPV genotype infection by examining different populations, including those with and without hrHPV infection, some studies have found the risk of developing HSIL with hrHPV infection type (e.g., HPV 31, 33, and 45) to be equal to or greater than that for hrHPV 18^24^. More recent developments in HPV screening strategies and genotype-specific risk assessments have led to recommendations emphasizing individualized risk assessment^25^. Collectively, these data suggest that more prospective studies are needed to establish the best treatment options for women with non-hrHPV infection but with normal cervical cytology.

### Strengths and limitations

This study's strength lies in its prospective design, which minimizes recall bias, and in its ability to accurately compare women who underwent colposcopy at the first visit with those who did not. Furthermore, CE were conducted by an experienced examiner to ensure procedural consistency and reduce interobserver variability. The presence of a follow-up colposcopic examination and cotesting at 1 year provided additional support for the validity of this study's findings.

There are, however, some limitations to be considered. First, because this study was conducted at a single tertiary center, its generalizability to other populations may be limited. Second, although the sample size was adequate for a preliminary assessment, it was relatively small for subgroup analyses. The probability of a Type II error could have been reduced by increasing the sample size. Third, HPV genotyping beyond HPV subtypes 16 and 18 could not be stratified into individual subtypes, which could provide a more precise risk assessment for individual patients. Fourth, a 1-year follow-up period may not give a complete picture of the long-term progression of cervical lesions; larger multicenter studies with longer durations of follow-up are necessary.

## CONCLUSION

This research investigated the effectiveness of early colposcopy in women with positive hrHPV results but normal cytology compared with traditional follow-up. The results indicate that 1 year after the initial diagnosis, no significant differences were observed between the two groups in hrHPV persistence, rates of abnormal cytology, or rates of LEEP use. Early colposcopy has enabled the treatment of HSIL lesions without increasing the number of LEEP procedures. Notably, 1 year after diagnosis, women who underwent early colposcopy had no high-grade lesions (HSIL) detected, compared with limited HSIL detection in women who did not undergo early colposcopy. It is expected that prompt treatment of the lesions will lead to a reduction in their number 1 year later. However, there is insufficient data to determine whether a 1-year delay leads to progression. Due to the limitations of the study design/sample size, these results are not conclusive regarding whether early colposcopy is superior to traditional follow-up and should therefore be interpreted with caution. The diagnosis of cervical adenocarcinoma in one woman who did not receive early colposcopy is a noteworthy clinical observation; however, it cannot be generalized to the entire cohort based on a single instance. As such, there is no evidence to endorse routine use of early colposcopy as a follow-up on women with normal cytology alone found to have positive hrHPV status. The findings of this study suggest that the risk-based follow-up approach remains a valid treatment option for this patient group. Additional large-sample studies or long-term prospective studies will also help definitively determine this.

## Data Availability

The datasets generated and/or analyzed during the current study are available from the corresponding author upon reasonable request.
